# Dopamine as an endogenous regulator of innate immunity in sepsis

**DOI:** 10.3389/fimmu.2025.1625368

**Published:** 2025-07-09

**Authors:** Haichao Wang, Rui Kang, Daolin Tang

**Affiliations:** ^1^ The Feinstein Institutes for Medical Research, Northwell Health, Manhasset, NY, United States; ^2^ Departments of Emergency Medicine and Molecular Medicine, Donald and Barbara Zucker School of Medicine at Hofstra/Northwell, Hempstead, NY, United States; ^3^ Department of Surgery, University of Texas (UT) Southwestern Medical Center, Dallas, TX, United States

**Keywords:** dopamine, sepsis, aconitate decarboxylase 1, CD274, innate immunity

## Abstract

Sepsis, a life-threatening organ dysfunction caused by a dysregulated host response to infection, presents a major clinical challenge. While the complex interplay of inflammatory mediators and immune cells during sepsis is increasingly understood, the role of neurotransmitters, particularly dopamine, in modulating the innate immune response is emerging as a crucial area of investigation. Dopamine, traditionally recognized for its role in the central nervous system, acts as an endogenous regulator of innate immunity, significantly influencing the course and outcome of sepsis. In this mini-review, we highlight our recent finding of dopamine’s critical role in regulating aconitate decarboxylase 1 (ACOD1) in sepsis.

## Introduction

Sepsis is a life-threatening condition that arises when the body’s response to infections triggers a dysregulated production of various inflammatory cytokines (1). It accounts for almost 20% of total deaths worldwide (2), and annually cost >$60 billion in the U.S. alone. Animal models remain indispensable in sepsis research, providing a controlled environment to unravel complex pathophysiological mechanisms, identify therapeutic targets, and evaluate novel interventions (3). The intricate interplay of various innate immune cells, inflammatory mediators, and signaling pathways contributes to the pathogenesis and progression of sepsis (4, 5). While the inflammatory cascade initiated by the innate immune system is crucial for pathogen clearance, its dysregulation can lead to detrimental systemic inflammation and subsequent immunosuppression (6, 7). Understanding the intricate mechanisms governing this immune response is critical for developing effective therapeutic strategies.

Dopamine (DA), traditionally recognized for its role in the central nervous system (8), acts as an endogenous regulator of innate immunity through a complex interplay with various immune cells expressing dopamine receptors, primarily D1-like (DRD1 and DRD5) and D2-like (DRD2, DRD3, and DRD4) receptors (9), thereby significantly influencing the course and outcome of sepsis. Depending on the receptor subtype and the specific context, dopamine can trigger intracellular signaling cascades that can either enhance or suppress immune cell activities.

## Dopamine as a bridge between the nervous and innate immune systems

Dopamine (DA) is a central nervous system (CNS) neurotransmitter involved in the control of several key functions, such as cognition and movement. In the periphery, DA is produced by neuroendocrine cells, the adrenal glands, and neuronal fibers, and influence functions like blood pressure, sodium balance and adrenal and renal functions (10, 11), as well as glucose homeostasis and body weight (12). In addition to its primary role in the CNS, DA is now recognized as a key modulator of the innate immune response in sepsis. For instance, dopamine is produced and released by various types of immune cells including lymphocytes, macrophages, peripheral blood mononuclear cells (PBMCs), and dendritic cells (8, 13–17) during inflammation (18). This localized production suggests dopamine’s involvement in modulating immune responses through paracrine or autocrine signaling, regulating both neurological and immunological responses (16, 19, 20).

It is plausible that dopamine release is tightly regulated and triggered by specific immunological stimuli, such as pathogen-associated molecular pattern molecules (PAMPs) or damage-associated molecular pattern molecules (DAMPs). Understanding the temporal dynamics of dopamine production—when and for how long it is released in response to specific immune challenges—is crucial. Furthermore, different tissues might exhibit varying abundances of these cells, leading to tissue-specific effects of dopamine signaling. Therefore, a deeper understanding of this interplay between temporal and spatial dynamics of dopamine production is essential for developing targeted therapies that effectively harness the immunomodulatory potential of dopamine signaling in immune-related diseases. This includes further research into the precise regulation of dopamine release, variations in its production across different immune cell types and tissues, and the intricate relationship between neuronal and immune-derived dopamine.

DA interacts with D1-like (DRD1, DRD5) and D2-like (DRD2, DRD3, DRD4) receptors on immune cells, triggering specific intracellular signaling cascades and influencing immune cell activity depending on receptor subtype and the specific immune cell involved. For instance, DA modulates innate immunity, in part, by influencing neutrophil functions. As the first line of defense against invading pathogens, neutrophils are crucial for bacterial clearance. Expressing DRD3, DRD5, and to a lesser extent, DRD2 and DRD4 dopamine receptors (21), neutrophils are responsive to dopamine signaling. For instance, acting via D1-like receptors, DA inhibits neutrophil chemotaxis and phagocytosis (22), potentially mitigating excessive inflammation and tissue damage (23). While this inhibition may be beneficial in early sepsis by attenuating an overwhelming inflammatory response, prolonged suppression of neutrophil activity can compromise bacterial clearance and increase the risk of secondary infections. Furthermore, DA reduces neutrophil activity by decreasing endothelial adherence, reactive oxygen species and cytokine production (18), and impairing cell migration and phagocytosis (22, 24–27).

Human monocytes predominantly express DRD2 and DRD3, with lower expression of DRD4 and DRD5 (21). Consequently, DA suppresses LPS-mediated NF-κB activation and cytokine production in these cells (28). Additionally, DA modulates macrophage polarization towards the M2 phenotype, which contribute to tissue repair and resolution of inflammation (9), suggesting a potential role for DA in the later stages of sepsis by fostering an anti-inflammatory action. *In vivo*, DA and its agonists suppress inflammatory responses in mice, reducing LPS-induced production of IL-12p40 (29), TNF (30), IFN-γ, and nitric oxide in macrophages (31) primarily via D2-like receptors (DRD2/DRD3/DRD4) (32). Conversely, DA stimulates the production of anti-inflammatory cytokines, such as IL-10, in macrophages (32, 33), mounting an anti-inflammatory response.

## Therapeutic potential of dopamine-based agents

DA exerts cardiovascular effects by acting on α- and β-adrenergic receptors, increasing cardiac output, systemic vascular resistance, and blood pressure (34), thereby counteracting the hypotension and hypoperfusion characteristic of organ dysfunction. Consequently, DA is often a first-line vasopressor in sepsis and septic shock during overwhelming immune responses to bacterial infections (35). While both DA and norepinephrine (NE) are commonly used as first-line vasopressors in the treatment of septic shock (36–39), NE may demonstrate superior efficacy in clinical settings (37, 40).

*In vivo*, pharmacological DA administration modulates the secretion of hormones such as prolactin (41, 42), restores hepatic blood flow (43), and improves hemodynamics by increasing blood pressure/flow and causing vasodilatation (34). DA suppresses systemic inflammation by blocking the TRAF6/NF-κB pathway via a DRD5 receptor-mediated signaling axis involving ARRB2 and PP2A (44). Consistently, a dopamine D1-like receptor-specific agonist improves survival of septic mice, partly by inhibiting TNF, IL-1β (45), IL-6, and IFN-γ (46). Similarly, electroacupuncture of the sciatic nerve increases adrenal DA production in mice, which subsequently acts on DRD1 receptors to reduce systemic inflammation and protect against lethal sepsis (47). Clinically, low-dose DA may benefit splanchnic blood flow and oxygen consumption in patients with septic shock (48), aligning with the potential therapeutic benefits of dopaminergic agonists in septic diabetic patients by controlling both hyperglycemia and systemic inflammation (49). However, high-dose DA, compared to norepinephrine, is associated with increased arrhythmic events and mortality (50–52). Independent of norepinephrine (NE) use, DA administration is associated with higher mortality (51, 53) and a greater incidence of arrhythmic events compared to NE administration (40).

While the role of dopamine in modulating immune responses is increasingly recognized, it is presently unclear whether systemic and locally produced dopamine exert distinct effects on immune function during sepsis. Addressing this important question is crucial for refining our understanding of sepsis pathophysiology and developing targeted therapeutic interventions. As aforementioned, systemic dopamine, primarily derived from the nervous system, circulates throughout the body and can interact with dopamine receptors expressed on various immune cells. These interactions can modulate immune cell activity, including cytokine production, phagocytosis, and lymphocyte proliferation. In the context of sepsis, extensive evidence indicates that DA might play a protective role by modulating inflammatory responses (43, 46, 54). In contrast to systemic dopamine, locally produced dopamine is synthesized and released by immune cells themselves, acting within the immediate microenvironment. This localized production allows for precise and targeted modulation of immune responses within specific tissues or at the site of infection, influencing the activity of neighboring immune cells.

The distinct effects of systemic versus local dopamine in sepsis may stem from several factors. First, the concentration of dopamine at the site of action may differ significantly. Locally produced dopamine can achieve high concentrations within the immune microenvironment, potentially exceeding those achieved by circulating dopamine. Second, the specific dopamine receptor subtypes expressed on different immune cell populations and within different tissues may vary, leading to diverse downstream effects. Finally, the interplay between dopamine and other signaling molecules present in the local microenvironment, such as cytokines and chemokines, could further influence the net effect of dopamine on immune function. Therefore, disentangling the roles of systemic and locally produced dopamine in sepsis requires sophisticated experimental approaches. For instance, studies using conditional knockout mice, where dopamine production is selectively ablated in specific cell types or tissues, could help elucidate the distinct contributions of systemic and local dopamine. Furthermore, *in vitro* studies using co-culture systems of immune cells and other relevant cell types, such as endothelial cells, can provide valuable insights into the interplay between dopamine and other signaling pathways within the immune microenvironment.

Therefore, understanding the differential effects of systemic versus locally produced dopamine in sepsis has significant implications for developing targeted therapeutic strategies. Manipulating dopamine signaling pathways could offer novel approaches to modulating immune function and improving outcomes in sepsis patients. For instance, selectively enhancing local dopamine production by immune cells at the site of infection could promote bacterial clearance and dampen excessive inflammation. Conversely, modulating systemic dopamine levels or targeting specific dopamine receptor subtypes might be beneficial in mitigating the systemic inflammatory response and preventing organ damage. Therefore, deciphering the distinct roles of systemic and locally produced dopamine in sepsis is a critical area of future research. This knowledge will not only enhance our understanding of the complex immunopathology of sepsis but also pave the way for developing innovative therapeutic strategies that harness the immunomodulatory potential of dopamine signaling.

## Novel role of DA in the regulation of ACOD1 expression

**Figure 1:**
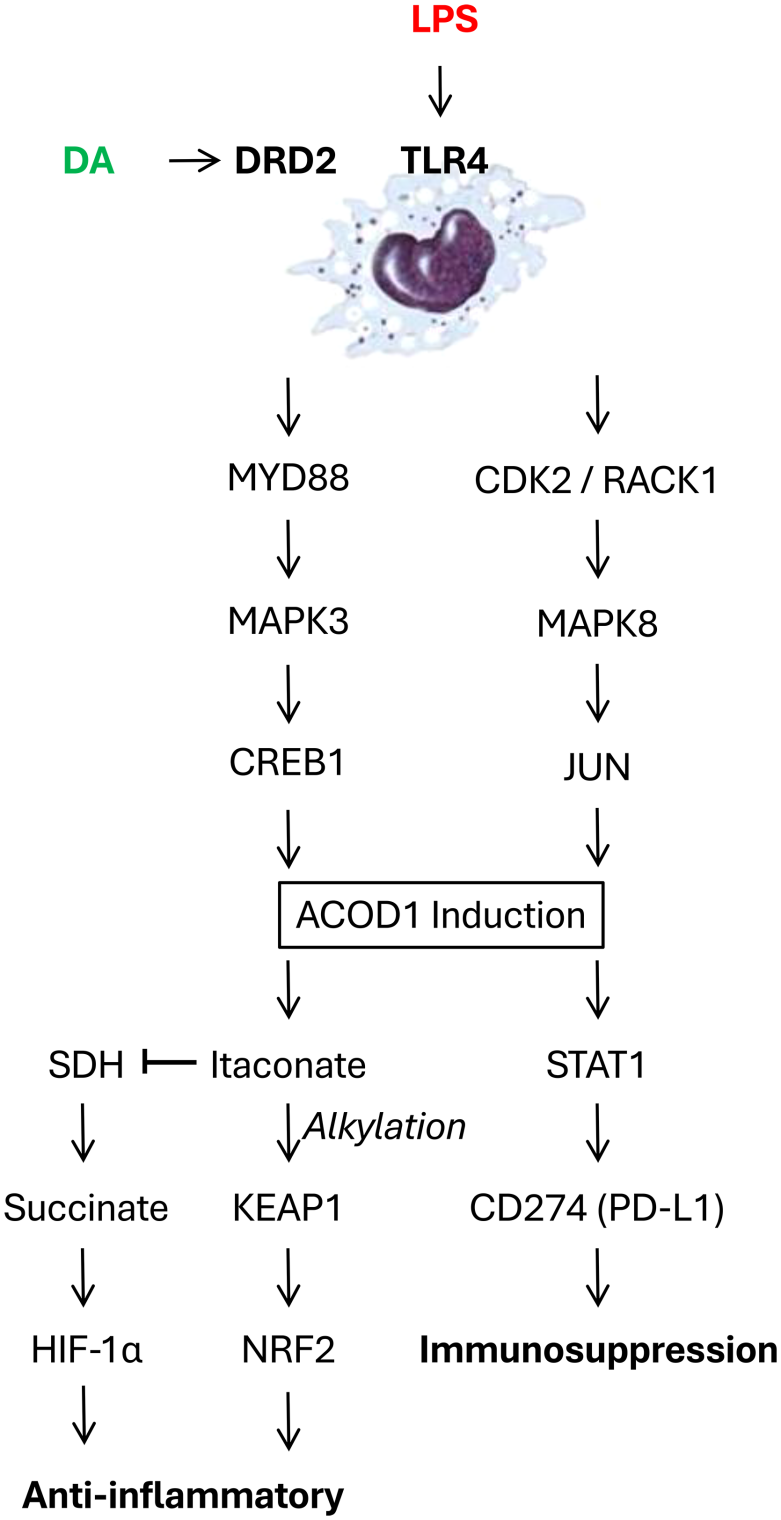
Dopamine (DA) counter-regulates bacterial endotoxins (LPS)-induced aconitate decarboxylase 1 (ACOD 1) expression in innate immune cells. LPS engages Toll-like receptor 4 (TLR4), activating either the MYD88-MAPK3-CREB1 or CDK2-RACK1-MAPK8-JUN signaling pathway, ultimately upregulating ACOD1 expression in innate immune cells. This ACOD1 upregulation increases itaconate production, which exerts anti-inflammatory effects by promoting direct alkylating Kelch-like ECH-associated protein 1 (KEAP1), activating nuclear factor erythroid 2-related factor 2 (Nrf2) and driving antioxidant and anti-inflammatory responses. Concurrently, ACOD1 upregulation also competitively inhibits succinate dehydrogenase (SDH), resulting in succinate accumulation which stabilizes hypoxia-inducible factor-1α (HIF-1α) and promotes anti-inflammatory gene expression. Furthermore, ACOD1 can promote the expression of the immune checkpoint inhibitor CD274 (PD-L1) via STAT1 activation. The engagement of DRD2 by dopamine or agonists can disrupt TLR4-MYD88 interaction, inhibit ACOD1 expression, thereby conferring protection against lethal sepsis.

Aconitate decarboxylase 1 (ACOD1, also known as immune-responsive gene 1, IRG1) is a critical regulator of immunometabolism and inflammation, particularly in the context of infection and injury. While initially recognized for its role in generating the anti-inflammatory metabolite itaconate, ACOD1’s functions have proven to be multifaceted, encompassing both itaconate-dependent and -independent mechanisms (**Figure 1**). Initially, the well-characterized function of ACOD1 relates to its catalysis of cis-aconitate to itaconate within the mitochondria. This activity is markedly upregulated in macrophages and other immune cells upon stimulation with inflammatory stimuli like lipopolysaccharide (LPS) (55). Itaconate, in turn, exerts a range of anti-inflammatory effects through multiple mechanisms such as: 1) competitive inhibition of succinate dehydrogenase (SDH), leading to succinate accumulation and stabilization of hypoxia-inducible factor-1α (HIF-1α) which promotes anti-inflammatory gene expression (56); 2) direct alkylation of proteins like Kelch-like ECH-associated protein 1 (KEAP1), resulting in activation of nuclear factor erythroid 2-related factor 2 (Nrf2) and subsequent antioxidant and anti-inflammatory responses (**Figure 1**) (57); and 3) inhibition of glycolysis, contributing to the metabolic reprogramming of activated immune cells (58). Collectively, through these mechanisms enable itaconate to dampen inflammation and promotes tissue repair.

Our recent research has uncovered itaconate-independent functions of ACOD1 (59), adding complexity to its immunoregulatory role in sepsis. Specifically, an LPS-stimulated, JUN-regulated, pro-inflammatory function of ACOD1 has been identified, involving an interplay between CDK2, RACK1, and MAPK8 (59). Mechanistically, LPS triggers the formation of a CDK2-RACK1-MAPK8 complex, leading to MAPK8 activation and JUN phosphorylation (**Figure 1**). Phosphorylated JUN then translocates to the nucleus and promotes ACOD1 expression (**Figure 1**) (59, 60). In a mouse model of sepsis induced by cecal ligation and puncture (CLP), global or myeloid-specific genetic knockout of either CDK2 or ACOD1 significantly improved survival (59), attenuating systemic inflammation, organ dysfunction, and coagulopathy. These protective effects have been observed even in the absence of itaconate production, supporting the existence of itaconate-independent mechanisms. Pharmacological inhibition of CDK2, a kinase upstream of ACOD1, with dinaciclib has replicated these benefits in CLP and other clinically relevant sepsis models (e.g., *E. coli* and *S. pneumoniae*) (59), suggesting that targeting the CDK2-ACOD1 axis may be a promising therapeutic strategy.

Recently, we have identified a key role for DA in regulating ACOD1 expression through a comprehensive screening of neurotransmitters for their ability to modulate LPS-induced expression of ACOD1 (54). Dopamine’s inhibitory effect was observed at both the mRNA and protein levels, suggesting a mechanism of transcriptional regulation. Furthermore, we pinpointed DRD2 as the specific receptor mediating dopamine’s inhibitory effect on ACOD1 expression (**Figure 1**) (54). Consistently, DRD2 knockdown and knockout reversed dopamine’s suppression on ACOD1 expression, while the DRD2 agonist ropinirole mimicked dopamine’s effect (54). These findings establish DRD2 as a crucial receptor in mediating DA’s immunomodulatory function in sepsis, opening avenues for targeted therapeutic interventions.

To elucidate the molecular mechanism underlying dopamine-mediated suppression of ACOD1 expression, we assessed the potential involvement of the TLR4-MYD88-MAPK3-CREB1 signaling pathway. A transcription factor, CREB1, was identified as a critical regulator of ACOD1 expression (54). Dopamine, acting through DRD2, inhibits CREB1 phosphorylation at Ser133, thereby suppressing ACOD1 transcription (**Figure 1**). Upstream of CREB1, the canonical TLR4-MYD88-MAPK3 pathway also plays a central role, with dopamine disrupting the interaction between TLR4-MYD88 interaction, leading to decreased MAPK3 activation and subsequent reduction in CREB1 phosphorylation (**Figure 1**) (54).

While ACOD1 is known for its role in producing the anti-inflammatory metabolite itaconate (61), our study also revealed an itaconate-independent function of ACOD1 in regulating the expression of CD274 (PD-L1), a crucial immune checkpoint inhibitor (**Figure 1**). ACOD1 promotes CD274 expression via STAT1 activation (54). Consequently, dopamine, by inhibiting ACOD1 upregulation, indirectly suppresses CD274 expression. This finding has significant implications for understanding the immunosuppressive phase of sepsis, as CD274 contributes to T-cell exhaustion and dysfunction. This itaconate-independent role of ACOD1 highlights its multifaceted involvement in immune regulation. At present, it is entirely unknow whether DA inhibits CD274 expression partly by inhibiting itaconate-independent activity of ACOD1.

We also explored the therapeutic potential of modulating DA signaling in sepsis. Pramipexole, a DRD2 agonist, conferred a significant protection in mouse models of endotoxemia and polymicrobial sepsis (54). Even when administered after the onset of sepsis, pramipexole significantly improved survival rates, reduced pro-inflammatory cytokine levels, attenuated organ damage, and downregulated ACOD1 and CD274 expression (54). These results suggest that enhancing dopamine signaling through DRD2 agonism, specifically using pramipexole, could represent a promising therapeutic strategy for sepsis.

These preclinical findings were corroborated by clinical data from sepsis patients. Non-survivors exhibited lower circulating dopamine levels and higher ACOD1 expression in peripheral blood mononuclear cells compared to survivors (54). This inverse correlation between dopamine and ACOD1 expression in human sepsis underscores the clinical relevance of our animal studies. The observed association of ACOD1 and CD274 with inflammatory markers in patients further reinforces the potential role of this axis in sepsis pathogenesis (54). However, larger and well-controlled clinical trials are needed to evaluate the efficacy and safety of DRD2 agonists, like pramipexole, in diverse sepsis patient populations, considering factors like sepsis stage, infection source, and comorbidities.

## Conclusions

Our recent studies have confirmed a crucial immunoregulatory role for dopamine in sepsis via the DRD2-TLR4-ACOD1-CD274 axis. Dopamine, acting through DRD2, inhibits the TLR4-MYD88-MAPK3 pathway, suppressing CREB1 phosphorylation and downregulating ACOD1 (**Figure 1**). This, in turn, impacts both the inflammatory and immunosuppressive phases of sepsis, influencing cytokine production, and ultimately animal survival. While these new findings offer promising new avenues for sepsis treatment, further investigation is crucial to translate these findings into clinical practice. It will be important to fully elucidate the complex interplay of dopamine and innate immunity in sepsis, including the roles of specific dopamine receptor subtypes, dopamine production by immune cells, and its impact on distinct immune cell subsets. A more comprehensive understanding of dopamine’s multifaceted effects on the innate immune response during sepsis is essential for developing effective therapeutic strategies.
